# Organosolv Lignin Improved Thermoplastic Elastomeric Behavior of Polyethylene/Polyisoprene Blend

**DOI:** 10.1021/acsomega.1c06062

**Published:** 2022-03-01

**Authors:** Arun Ghosh

**Affiliations:** Center for Materials & Manufacturing Sciences, Department of Chemistry & Physics, Troy University, Troy, Alabama 36082, United States

## Abstract

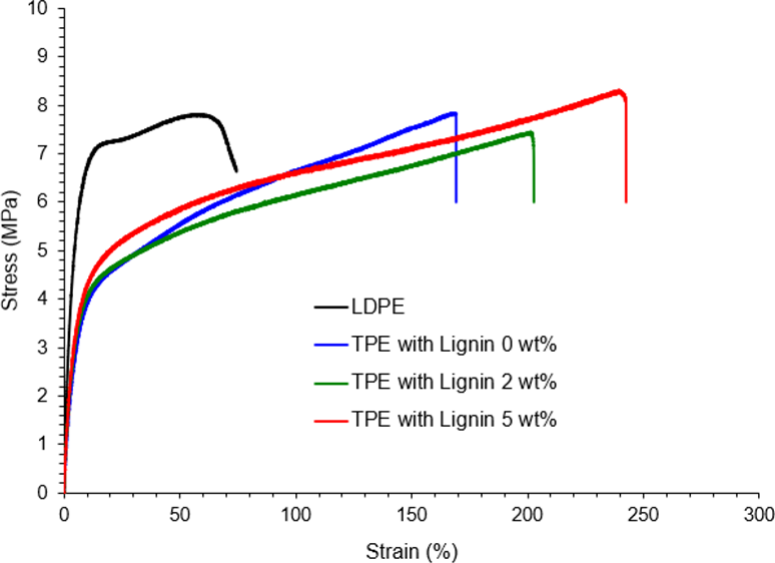

Thermoplastic elastomers are considered the fastest-growing elastomers in recent years because of their thermomechanical recyclability, in contrast to traditional thermoset rubbers. Polyolefins such as low-density polyethylene (LDPE) show low mechanical properties, particularly poor elongation when compared with an elastomer or rubber. In this study, LDPE resin is converted to highly ductile rubber-like materials with high elongation and low modulus properties on blending with polyisoprene rubber (IR), followed by treating with dicumyl peroxide as a curing agent and organosolv lignin as an additive. The technique of high shear melt-mixing, in conjunction with vulcanization or crosslinking using organic peroxide, is used to develop hybrid materials based on the LDPE/IR blend at a 70/30 mass ratio, where LDPE is replaced partly with lignin. Various characteristics such as tensile, viscoelasticity, melt flow, crystallinity, and phase morphology of the materials are analyzed. As expected, vulcanization with peroxide can improve the mechanical performance of the LDPE/IR blends, which is further improved with the application of lignin (2 to 5 wt. %), particularly tensile strain is profoundly increased. For example, the average values of the tensile strength, the modulus, and the ultimate elongation of neat LDPE resin are 7.8 MPa, 177 MPa, and 62%, respectively, and those of LDPE/IR/lignin/DCP 65/30/05/2 are 8.1 MPa, 95 MPa, and 238%, respectively. It indicates that the application of lignin/DCP has a profound effect on improving the ductility and elastomeric characteristics of the materials; thus, this material can have the potential to replace traditional rubber products.

## 1 Introduction

Thermoplastic elastomers (TPEs) exhibit mechanical properties that are comparable to those of traditionally vulcanized thermoset rubbers. Importantly, TPEs possess thermally reversible crosslinked networks that make them suitable for melt-processing using techniques such as melt-compounding, extrusion, and injection molding.^1,2^ According to the molecular compositions, TPEs are broadly classified into two groups: block copolymers and thermoplastic/elastomer blends. This class of polymers is expected to have the following two main characteristics: (a) the ability to be stretched to moderate elongations and, upon the removal of stress, return to something close to its original shape and (b) processable as a melt at elevated temperature. TPEs like materials have found applications as adhesives, elastomers, coatings, fibers, and feedstock for additive manufacturing, in preparing items starting from our daily life products to materials for use in building construction, medical devices, and many other advanced systems.^3,4^ In recent years, TPEs are considered the fastest-growing elastomers to replace traditional unrecyclable thermoset rubbers because of environmental protection and resource-saving.^5^ In 2015, the global market of TPEs was over USD 12 billion, which is estimated to be worth over USD 20 billion by 2023.^4^

TPE-like materials consist of two or more polymeric phases, with one phase that is hard at room temperature but becomes fluid at high temperatures, and the other phase is discontinuous, which is soft and elastic at room temperature.^5^ The hard phase provides physical crosslinked structures, and the soft phase acts as a rubbery matrix. In several TPEs, such as polystyrene-based block polymer or blends, the rigid polystyrene unit acts as a hard segment. For example, polystyrene is the hard phase and provides a physical crosslink in the polystyrene/natural rubber blend.^6^ In addition, there are several reports of producing TPEs based on graft or copolymerization techniques.^7,8^ Materials with TPE-like characteristics can also be prepared from several blends of plastics and elastomers using the melt-compounding technique. The application of dynamic vulcanization using an organic peroxide can improve certain characteristics of TPEs, including the permanent set, ultimate mechanical properties, resistance to chemical attack, high-temperature utility, stability of the phase morphology, melt strength, and more reliable thermoplastic fabricability.^9^ The TPEs produced using the blends of rubbers and plastics are advantageous when considering the reprocessing and performance of the materials. The modification of a TPE’s properties is achieved via simple techniques such as the variation of the blend composition and compounding ingredients and conditions.^2^ Several polyolefin-based TPEs were produced using different blends such as blends of high-density polyethylene with nitrile rubber,^9−11^ epoxidized natural rubber,^12^ ethylene–vinyl acetate,^13^ blends of polypropylene with ethylene–propylene rubber,^14^ epichlorohydrin rubber,^15^ styrene–butadiene rubber,^16^ fluorocarbon rubber,^17^ styrene–ethylene butadiene styrene block polymer,^18,19^ ethylene–octene copolymer,^20^ acrylic rubber,^21^ and blends of polyethylene with silicone rubber,^22^ ethylene-1-octene copolymer,^23^ and ethylene–propylene rubber.^24,25^ Typically, TPEs show the high elasticity of traditional vulcanized rubber and the good thermal processability and recyclability of classical thermoplastics. It is anticipated that the substitution of traditional vulcanized rubber by TPEs can enhance productivity and save energy and resources.^5,26^

Lignin, consisting of polyphenolic units, is a major component of lignocellulosic plants. Lignin has a globular, rigid, and nanoscale structure with the dimension of a few nanometers and a molecular weight of a few hundred to a few thousand Daltons. Thus, lignin has the potential to significantly improve the TPE behavior of a polymeric material.^27^ Among the different extraction techniques, the organosolv process produces lignin chemicals with less sulfur crosslinked and salt-free structures with high purity.^28−30^ Particularly, lignin extracted from softwood such as hybrid poplar tree via organosolv fractionation consists of high syringyl/guaiacyl monolignol ratios, which can lead to good melt-stability and flow behavior in the temperature range of 140 to 170 °C,^28,29^ similar to that of traditional polyethylene resins such as low-density polyethylene. Therefore, it is anticipated that this organosolv lignin can produce homogeneous blends with low-density polyethylene (LDPE) via the melt-compounding technique when appropriate compatibilizing or crosslinking additives are used. In addition, lignin has a relatively high glass transition temperature of over 110 °C because of its aromatic ring structures.^31^ Recently, there is progress in using lignin as a hard domain in making TPEs with different functional properties using graft copolymerization.^27,32−34^ Using a reactive blending technique, the lignin-based thermoplastic elastomeric blends were prepared with other functional polymers such as nitrile rubber,^35−37^ polyethylene glycol,^38^ and ethylene-1 octene copolymer.^39,40^ Interestingly, lignin has hydrogen transfer or donating ability because of its polyphenolic structures with reactive OH groups. Therefore, lignin has the potential to behave as a cocuring or coagent in rubber crosslinking with an organic peroxide. The addition of lignin-like multifunctional compounds has the ability to create more reactive sites within the resin matrix for effective crosslinking of a polymer chain with another polymer radical or addition reactions through in-chain or pendant double bonds. Such coagent compounds can eliminate or reduce the occurrence of competitive undesirable or destructive reactions involving polymer chain scission or other degradation during peroxide curing.^41^ In this study, LDPE is used in the processing of thermoplastic elastomers in conjunction with polyisoprene (IR) rubber. The TPE samples are prepared based on the LDPE/IR blend of the 70/30 mass ratio using dicumyl peroxide as a crosslinker and lignin as a coagent or performance modifier using the technique of high shear melt-compounding. The objective of this study is to use organosolv lignin as an additive for improving the thermoplastic elastomeric behavior of the LDPE/IR blends.

## 2 Results and Discussion

### 2.1 Mixing Torque and Temperature

**Figure 1:**
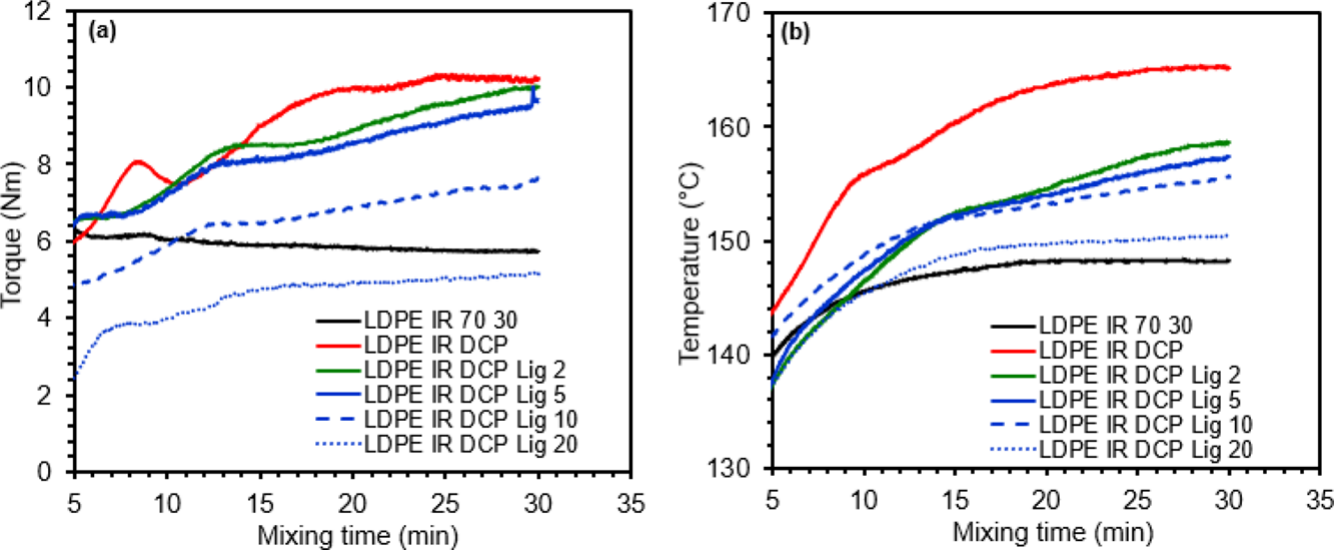
Mixing time vs torque (a) and temperature (b) during melt-compounding, showing the effect of lignin addition on mixing parameters.

The observed variations in mixing torque and temperature during high shear melt-compounding are related to some key characteristics of blending ingredients such as chemical reactivity and compatibility or crosslinking ability, molecular entanglement and interactions, plasticization, and melt viscosity. In this study, to produce a thermoplastic elastomer, LDPE was blended with polyisoprene rubber, DCP, and lignin (according to the formulation shown in Table 1) using the IntelliTorque Plasticorder, where the lignin content in the blends was varied from 0 to 20 wt. %. The changes in mixing torque and the temperature of the samples against time as recorded during mixing are represented in Figure 1. The mixing profile for the first 5 min is not shown in this report, which was associated with the feeding of the ingredients in the order of LDPE, IR, lignin, and DCP, as appropriate. In this initial period, sharp increments in mixing torques were observed, which is attributed to the resistance exerted by the unmolten resin granules during feeding into the mixer. After complete feeding of all ingredients into the mixer, there was a rapid decrease in the mixing torque because of the melting of all ingredients. In progress with blending, the mixing torque changed or remained constant, depending on the compositions of each blend. It appeared that the equilibrium torque of LDPE/IR (without any additive) is approximately 6 Nm, and during dynamic vulcanization of LDPE/IR blend with DCP, the mixing torque gradually increased and reached a constant value of 10 Nm at approximately 25 min. The high mixing torque is attributed to the interphase crosslinking between LDPE and IR, where the IR phase becomes crosslinked predominantly. The mixing was allowed to continue for a further 5 min to achieve uniform blending. Interestingly, lignin addition decreased the equilibrium mixing torque, which is attributed to the presence of the non-crosslinked lignin phase in the blends. As reported elsewhere,^28^ this organosolv lignin had a low melt-viscosity at elevated temperatures compared to LDPE resin; therefore, in the blends, non-crosslinked low-viscous lignin acts as a plasticizing agent and facilitates the mixing procedure by reducing the overall melt viscosity of the blends. The reduced mixing torque reflects less energy requirement when considering large-scale product manufacturing.

**Table 1:** Summary of Tensile Stress–Strain and Solubility Characteristics of the Blends^a^

sample descriptions		tensile properties			solubility in trichlorobenzene	
compositions (mass ratio, %)	sample ID	maximum tensile stress (MPa)	ultimate strain (%)	Young’s modulus (MPa)	insoluble mass (wt. %)	crosslinked LDPE and IR phases (wt. %)
LDPE/IR 100/00	LDPE	7.8 ± 0.5	62 ± 3.2	177 ± 15	NA	NA
LDPE/IR 70/30	LDPE IR	4.5 ± 0.1	55.5 ± 5.7	90 ± 9	NA	NA
LDPE/IR/DCP 70/30/2	LDPE IR DCP	7.7 ± 0.5	178 ± 16	76 ± 2	NA	NA
LDPE/IR/lignin/DCP 68/30/02/2	LDPE IR DCP Lig 2	7.8 ± 1.0	212 ± 70	92 ± 7	NA	NA
LDPE/IR/lignin/DCP 65/30/05/2	LDPE IR DCP Lig 5	8.1 ± 0.6	238 ± 38	95 ± 6	28.8 ± 0.3	25.1 ± 0.3
LDPE/IR/lignin/DCP 60/30/10/2	LDPE IR DCP Lig 10	6.1 ± 0.2	108 ± 28	102 ± 10	31.0 ± 2.0	23.3 ± 2.0
LDPE/IR/lignin/DCP 50/30/20/2	LDPE IR DCP Lig 20	4.7 ± 0.7	36 ± 26	139 ± 5	36.4 ± 2.7	22.2 ± 0.6

a “NA” indicates that data are not available as the samples dissolved in the solvent during the experiment.

Although all samples were blended at a starting set temperature of 140 °C, during blending, the actual mixing temperature of the samples gradually increased because frictional forces within resins increased, depending on the compositions of the materials. The changes in the mixing temperatures of the blends followed the trends of mixing torque changes. The LDPE/IR blend without any additive showed an equilibrium mixing temperature of 148 °C, which increased to the value of 165 °C in the presence of organic peroxide because of the onset of crosslinking in the blends. The addition of lignin to the blend significantly dropped the actual mixing temperature, for example, the addition of 2 wt. % lignin reduced the mixing temperature of the vulcanized blend to around 157 °C, indicating that non-crosslinked lignin plasticized the LDPE/IR blend. The increase in the temperature is due to the high frictional forces generated within the blends during mixing because of the onset of polymer crosslinking. DCP molecules are cleaved at approximately 150 °C and produce free radicals, which are available for reactions with polymer blends, particularly with unsaturated polymers or additives such as lignin-like compounds with reactive groups.^42^ The increases in the mixing torque and temperature are related to several factors such as the onset of chemical crosslinking, plasticization by non-crosslinked lignin, molecular chain entanglement, or physical frictional forces among different fragments in polymer blends. The crosslinked phases are resistant to melt flow or deformation, creating high frictional forces and consequently increasing the mixing temperature and torque during blending. The reduction of the mixing torque and temperature of the blends due to lignin addition is attributed to the plasticization effect of non-crosslinked lignin.

### 2.2 Tensile Stress–Strain Properties

Tensile stress–strain behavior is one of the key properties of polymers and their blends or composites for any technical applications. This mechanical behavior of a TPE based on the plastic/rubber blend depends on the various chemical characteristics of soft and rigid phases such as chemical compatibility, molecular sizes, interphase morphology, plasticization, and processing techniques and conditions. In general, plastic/rubber blends are considered as TPEs if they exhibit reasonably good tensile strength like plastics and high elongation at break (>100%) like rubbers.^15,17^

**Figure 2:**
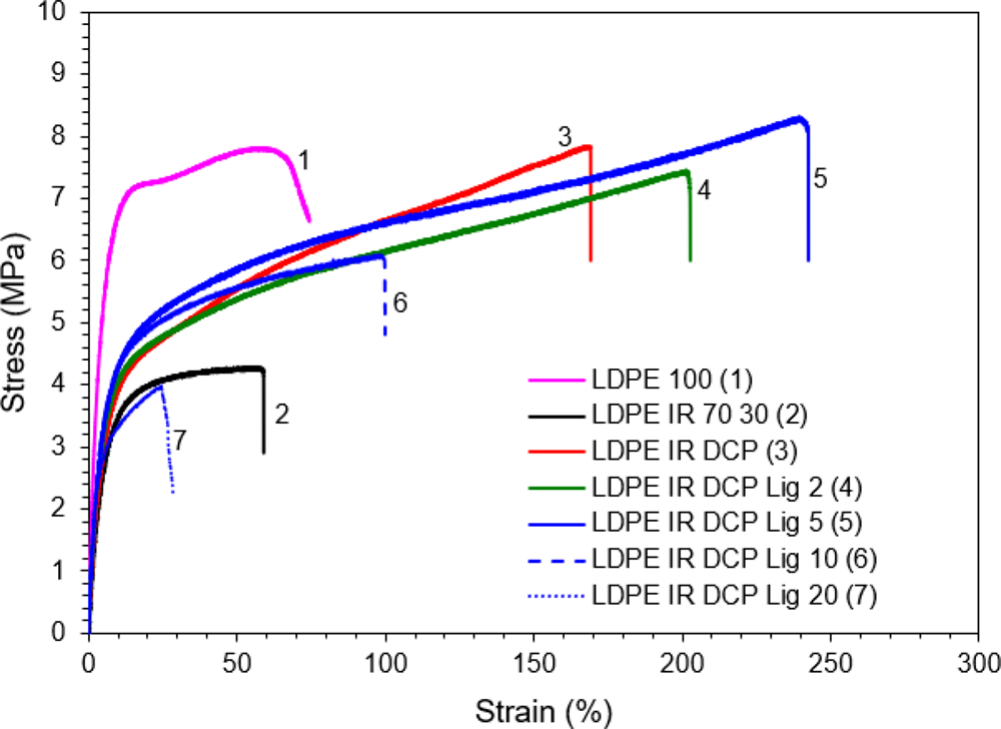
Representative tensile stress–strain plots of polyethylene/isoprene/lignin-based materials, showing that lignin (up to 5 wt. %) can improve the elastomeric characteristics of the materials.

Compared to thermoplastics such as polycarbonate or polypropylene, LDPE resin shows a relatively low range of mechanical properties. In this study, the virgin LDPE resin showed an average tensile strength of 7.8 MPa, Young’s modulus of 177 MPa, and an ultimate elongation of 62% (Table 1). Upon blending with uncured polyisoprene rubber, both tensile strength and modulus of the blends dropped significantly, which is attributed to the poor compatibility between LDPE and uncured IR phases because of differences in chemical makeup and melt-flow properties. Such differences in properties led to weak interfacial adhesion and poor stress transfer between the LDPE and IR phases during stretching, showing poor tensile characteristics. The application of dynamic vulcanization using organic peroxide during melt-compounding increased all of the tensile characteristics of LDPE/IR blends dramatically. The tensile behavior, particularly, tensile strain and ductility of the vulcanized blends were increased further upon the addition of lignin up to 5 wt. % concentration. For example, the 70/30 blend of LDPE/IR showed an average tensile strength of 4.5 MPa, a modulus of 90 MPa, and an ultimate strain of 56%, and the LDPE/IR blend crosslinked with DCP showed an average strength of 7.7 MPa, a modulus of 76 MPa, and an elongation of 178%, which were improved further upon the addition of lignin. Particularly, tensile elongation was improved significantly upon the addition of lignin, showing an average elongation of approximately 238% at a 5 wt. % concentration of lignin. The deterioration of tensile properties of the samples was observed upon further increasing the lignin content beyond 5 wt. % (Table 1). The representative stress–strain plots of various samples are presented in Figure 2. The lower tensile strength and strain of the materials containing a high amount of lignin (10% and more) are attributed to the presence of a more non-crosslinked lignin phase. Lignin is a low-molecular-weight (2262 Da) additive and mechanically fragile or brittle at ambient conditions^28^ when compared with the LDPE resin (135–163 kDa).

There are correlations between the tensile stress–strain properties and other characteristics such as the melt viscosity and the phase morphology with changing lignin contents in the blends. It is anticipated that because of the hydrogen-donating ability of polyphenolic structures,^43,44^ lignin can tune the crosslinking mechanism in the LDPE/IR blends. The radicals generated from DCP decomposition at elevated temperature (>150 °C) abstract hydrogen from lignin, resulting in lignin radicals available for crosslinking with LDPE and/or IR phases. Therefore, dynamically vulcanized LDPE/IR blends become crosslinked effectively with lignin during the molding process at 170 °C, increasing tensile strain and strength. In addition, because of irregular and bulky chemical structures, a small amount of (i.e., 5 wt. %) lignin increases molecular free volume in the LDPE/IR blends, resulting in increases in the tensile strain of the materials. Similar changes in tensile strength and strain were observed when nanoscale diamondoid molecules were added to a polypropylene resin, as reported elsewhere.^45^ The enhanced compatibility, which increased the maximum tensile strength and elongation at break values of the LDPE/IR blends in the presence of DCP and lignin, was also confirmed with the help of dynamic mechanical analysis (DMA) and morphological studies.

### 2.3 Dynamic Mechanical Analysis

The viscoelastic polymeric materials undergo phase change or transition because of the onset of molecular chain mobility when they are exposed to elevated temperatures. The inception of major phase transition associated with molecular chain motion during heating is considered as the glass transition (*T*_g_), above which polymers behave like rubbers. The viscoelastic characteristics such as glass transition and also tanδ (the ratio of loss and storage modulus) of polymers are measured using the technique of DMA. Tan δ is related to the damping or energy dissipation behavior and is associated with the total amount of energy absorbed by a material.^46^ A large area under the tanδ curve indicates a higher degree of molecular chain mobility and reflects a better damping behavior. The materials with high damping behavior are capable of absorbing and dissipating energy well, and they behave like highly ductile materials.

**Figure 3:**
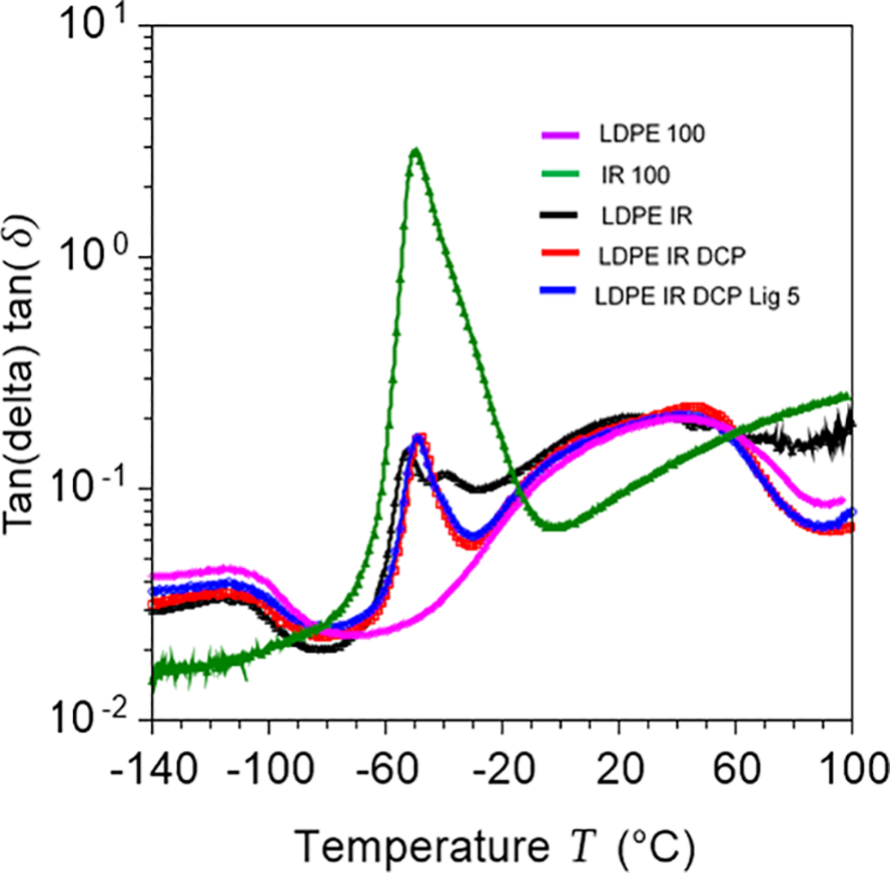
DMA profiles of LDPE, IR, and their elastomeric blends: temperature vs logarithm of tanδ, showing that lignin and DCP-modified blends have major glass-to-rubber transitions centered at −49 °C.

The viscoelastic profiles of polyethylenes were analyzed earlier, and three relaxation zones (usually designated as α, β, and γ in the order of decreasing temperature) were identified. α-relaxation is known to be involved with the crystalline phase and melting of polyethylene.^47,48^ In the present study, the key transition of LDPE was found at a low-temperature region centered at −104 °C (Figure 3), which is associated with the β-relaxation or glass transition of the LDPE resin. γ-relaxation reflecting the motion of a short polymer segment (e.g., three to four CH_2_) of the bulk amorphous fraction generally occurs in the range of −150 to −100 °C.^46,49^ In the current analysis, this γ-relaxation appeared to be merged with the β-relaxation or *T*_g_ of the LDPE resin.

The unmodified LDPE/IR blend showed a bimodal transition with two peaks centered at −52° and −40 °C, which are attributed to the phase transitions associated with polyisoprene domains in the blend. Neat IR rubber showed a glass transition peak centered at −50 °C (Figure 3). The previous studies also revealed that polyisoprene or natural rubber shows a glass transition peak centered at approximately −50 °C.^50^ In the present case, the bimodal transition of the IR phase in the LDPE/IR blend indicated that the blend is not homogeneously mixed. It is anticipated that in the unmodified blend, the peak at −52 °C is associated with the transition of isolated IR domains, whereas the peak at −40 °C is attributed to the IR moiety that is closely associated (entangled or encapsulated) with the LDPE phase. The close association with the LDPE matrix reduces the chain mobility of the IR chain, resulting in increased glass transition temperature of the local IR phase. However, after dynamic vulcanization, these two peaks merged and formed a single peak centered at −49 °C, indicating homogeneous crosslinking of the IR phase in the blend. The application of lignin did not further change the glass transition temperature or tan δ behavior of the blend.

### 2.4 Melt-Rheological Analysis

**Figure 4:**
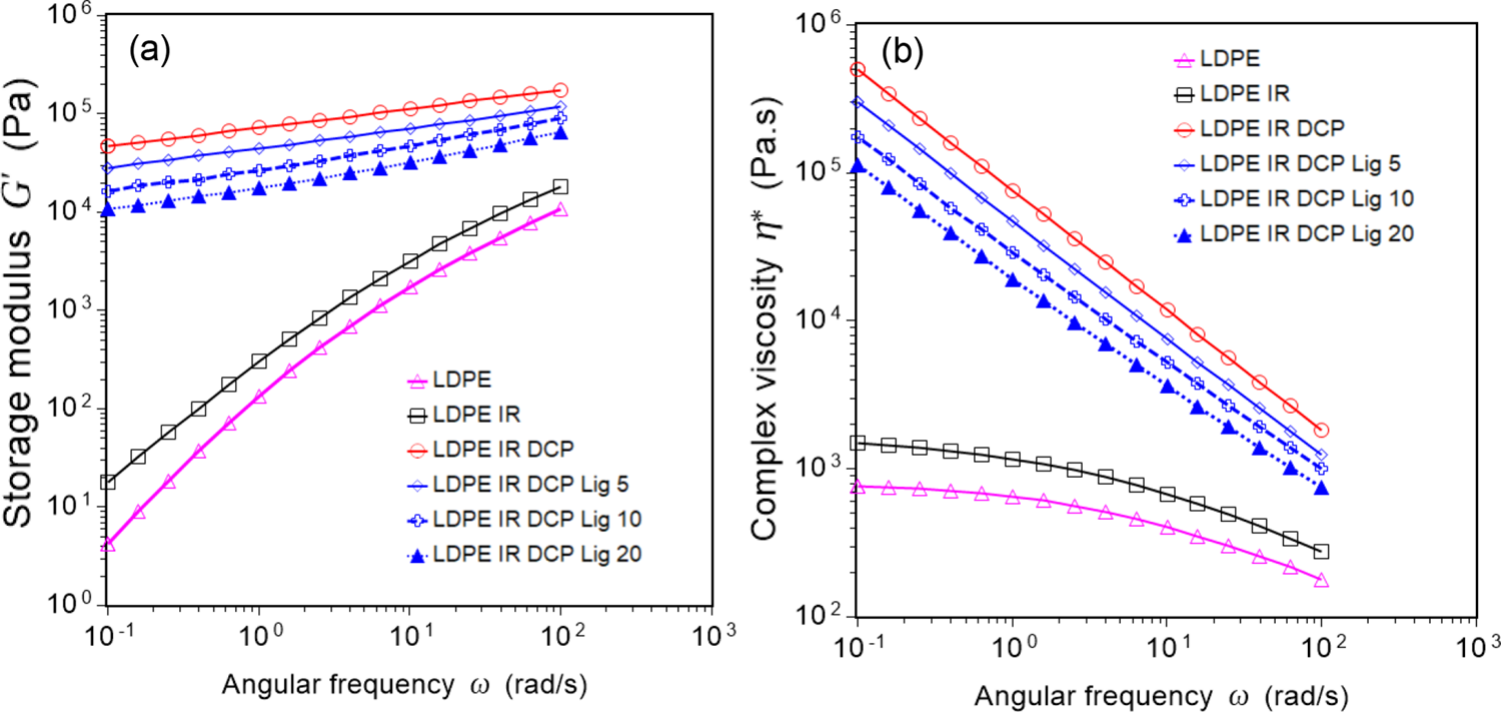
Melt-rheological behavior of LDPE and its blends: logarithm of angular frequency vs logarithm of storage modulus (a) and complex viscosity (b), showing that modified blends are relatively resistant to melt-flow but thermally melt-processable.

The melt-rheological data can provide an understanding of the thermal recyclability and melt-flow behavior of a thermoplastic or thermoplastic elastomer for the mass-scale production of any product. In the present study, the changes in interfacial crosslinking induced via dynamical vulcanization and lignin addition and subsequent melt-flow properties of the polymer blends based on LDPE/IR were investigated in terms of various rheological characteristics. The characteristics such as complex viscosity and storage modulus against the angular frequency (0.1 to 100 rad/s) of the blends and neat LDPE as measured at 170 °C are presented in Figure 4. Both the modulus and the viscosity of the modified blends were frequency-dependent: with increasing angular frequency (0.1 to 100 rad/s), the viscosity decreased and the modulus increased linearly, indicating that elastomeric blends are not strongly crosslinked thermosets like traditional rubbers.

**Figure 5:**
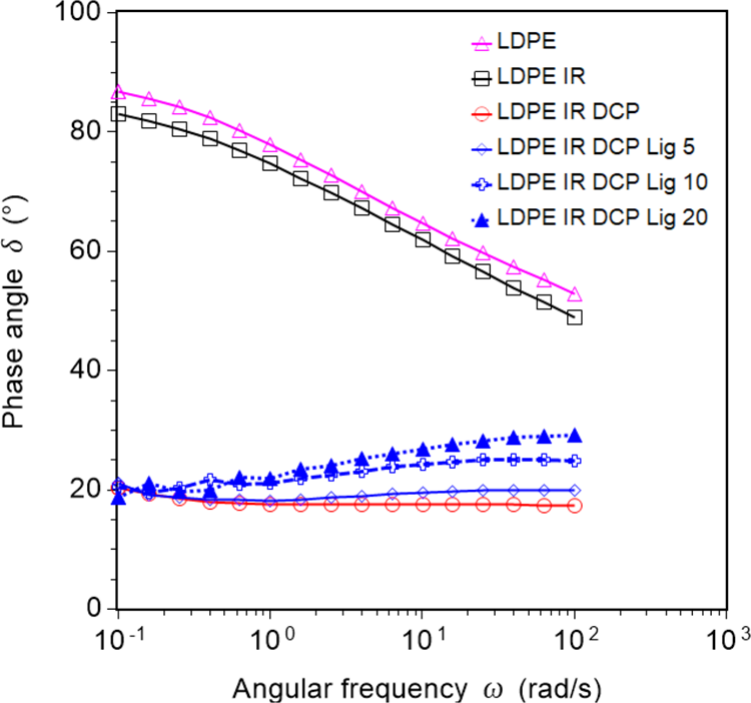
Phase angle against angular frequency of LDPE and its blends, showing that melts of modified blends have good phase recovery characteristics.

The storage modulus is a sensitive rheological function related to the structural changes in polymers. The melt storage modulus of all samples increased with increasing angular frequency. The neat LDPE and LDPE/IR blends showed a nearly similar storage modulus with a maximum value of approximately 10 to 20 kPa at 100 rad/s. In contrast, vulcanized blends showed significantly high storage modulus, for example, the peroxide cured blend (LDPE IR DCP) showed a maximum modulus of 180 kPa at 100 rad/s, which dropped linearly upon the addition of lignin. As expected, the complex viscosity values of all samples decreased with increasing angular frequency, but the viscosity values depend largely on the level of interfacial crosslinking in addition to the angular frequency. For example, at 1.0 rad/s, the neat LDPE and LDPE/IR blend exhibited a viscosity value of 650 and 1200 Pa.s, respectively, and vulcanized blends revealed a dramatically high complex viscosity, where the peroxide cured blend (LDPE IR DCP sample) showed a maximum viscosity of 110 kPa s at 1.0 rad/s, which dropped linearly upon the addition of lignin. The melt-flow resistance of the samples was also expressed as the phase angle against the angular frequency (Figure 5). The neat LDPE and LDPE/IR blend showed a high phase angle in the range of 50 to 87°, indicating low phase recovery on the withdrawal of shear stress. In contrast, the vulcanized blends exhibited a relatively low phase angle of approximately 20°, which increased upon increasing the lignin content in the blend. This also revealed that the melts of crosslinked blends are more elastic compared to neat blend and LDPE, indicating good phase recovery after the withdrawal of shear stress.

In brief, the melt-rheological analysis revealed that the crosslinked blends are resistant to melt-flow or elastic at elevated temperatures, as studied at 170 °C, which is attributed to the interfacial crosslinked structures in the blends. However, the steady change in the melt flow profile with the angular frequency indicated that the blends are not strongly crosslinked. Instead, they acted as thermoplastics, and therefore, they could be thermally recyclable. In summary, all blends exhibited shear-thinning of typical non-Newtonian fluids at elevated temperatures. These blends acted as classical thermoplastics and revealed thermal molding and reprocessing characteristics. Previous studies revealed that peroxide crosslinking can create thermoset elastomers, which do not melt, instead of degrading upon heating.^42,51^ In the present work, the organic peroxide, in conjunction with lignin, produced blends based on LDPE/IR with tunable melt-viscosity and storage modulus, but their rheological characteristics are angular frequency-dependent, indicating that the blends are not highly crosslinked thermoset; instead, they behave like a thermoplastic elastomer.

### 2.5 Change in Crystalline Behavior

**Figure 6:**
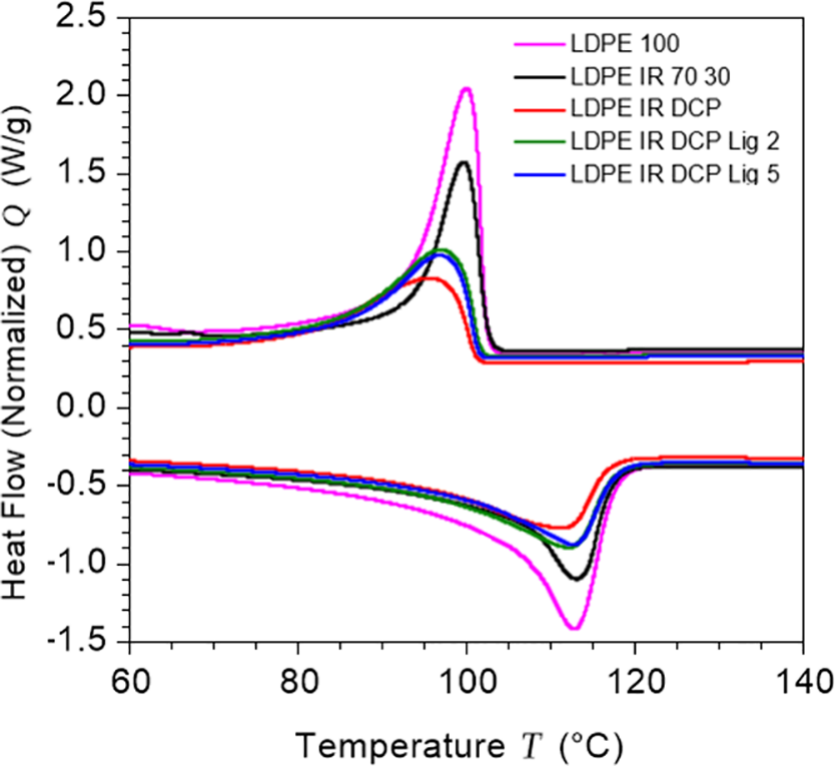
DSC plots of neat LDPE and LDPE/IR blends, showing decreases of crystallization enthalpies and melting enthalpies of the blends, while crystallinity of the LDPE phase is marginally changed.

The key thermal transition characteristics associated with the melting and crystallization of the crystalline phase of LDPE resin in the blends were analyzed using the DSC technique. Figure 6 represents the DSC melting and cooling cycles of neat LDPE and its blends without and with lignin, as determined under nonisothermal conditions. The cooling and melting parameters extracted from the DSC plots are summarized in Table 2. It appeared that neat LDPE and its blend with polyisoprene show a crystallization peak centered at 100 °C, which is lowered with the application of peroxide and lignin. This drop of the crystallization peak is attributed to the dispersion of the crosslinked LDPE phase in the blends, where the realignments of LDPE molecules are restricted. However, the onset temperature of the crystallization of neat LDPE at 102 °C remained almost unchanged upon blending with other additives. The crystallization enthalpy (Δ*H*_c_) of neat LDPE was 84 J/g, which dropped significantly upon blending with polyisoprene, and a further decrease in Δ*H*_c_ was observed after vulcanization and lignin addition. These changes in Δ*H*_c_ values suggested that the crosslinked phases in the blends can depress the rearrangement of LDPE chain segments during the crystallization process. The percent crystallinity (*X*_c_) and melting enthalpy (Δ*H*_m_) values of blended materials were also low when compared to the values of neat LDPE. This also clearly explained that the presence of additives such as isoprene rubber and lignin restricts the rearrangements of LDPE molecules into crystalline phases. Particularly, dynamic vulcanization reduced the crystalline nature of LDPE in the blend, which was regained slightly in the presence of lignin, as described in Table 2. It is anticipated that lignin acts as a hydrogen-donating agent, resulting in lignin radicals for crosslinking with LDPE/IR phases and eliminating undesirable polymer chain fragmentation reactions during dynamic crosslinking. Therefore, the LDPE phase in the blends appeared to exhibit higher crystallinity as the crosslinked LDPE phase does not contribute to crystallization. In addition, the increase in the crystallinity of the non-crosslinked LDPE phase in the blends may be attributed to the nucleation effect of lignin. There are several additives available in the markets that can increase the crystallinity of polyethylene products.^52^

**Table 2:** Thermal Transition Characteristics of Nonisothermally Crystallized Samples by DSC

sample	cooling cycle			melting cycle		
	onset crystallization temperature (°C)	peak crystallization temperature (°C)	crystallization enthalpy, Δ*H*_c_ (J g^–1^)	melting temperature peak (°C)	melting enthalpy, Δ*H*_m_ (J g^–1^)	percent of crystallinity (%)
LDPE	102	100	84	113	96	33
LDPE IR	102	100	55	113	63	31
LDPE IR DCP	101	96	45	112	55	27
LDPE IR DCP Lig 2	102	97	54	112	62	32
LDPE IR DCP Lig 5	101	97	50	113	57	30

### 2.6 Blend Morphology

**Figure 7:**
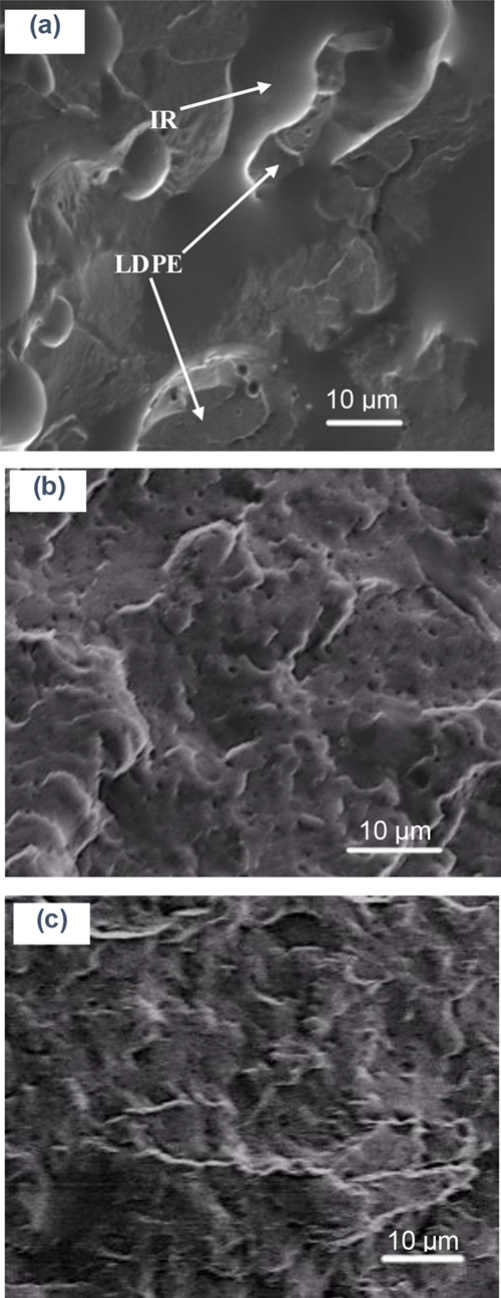
SEM photomicrographs of cryogenically fractured samples: (a) LDPE IR, (b) LDPE IR DCP, and (c) LDPE IR DCP Lig 5, showing the improvement of LDPE/IR blend compatibility with DCP and lignin.

The scanning electron microscopy (SEM) imaging technique is used for the qualitative investigation of the phase morphology of blends, which helps in predicting interfacial adhesion or chemical compatibility and thus the improvement in the mechanical performance of the polymer blends. In general, the blend morphology is largely dependent on the chemical characteristics and melt-viscosities of the constituent polymers, mass ratios of components, and melt-processing techniques.^53−55^ The SEM photomicrographs of cryogenically fractured surfaces of the neat and modified blends of LDPE/IR are shown in Figure 7. The SEM image showed that the uncured or neat blend of LDPE with the IR resin is incompatible and creates a coarse-phase morphology compared to the crosslinked blends. The two phases of LDPE and IR were distinguishable in the SEM image (Figure 7a), where the high viscous LDPE acts as a core or a dispersed phase and the low-viscous IR acts as a continuous coating phase. Notably, as expected, interfacial adhesion observed in the neat LDPE/IR blend was relatively better compared to the other blends of polyolefins with polar polymers such as polycarbonates, as reported elsewhere.^56−59^ However, in the present study, compatibilization was significantly improved for the LDPE/IR blend when the vulcanization technique was applied using DCP as a crosslinker, with or without lignin as an additive (Figure 7b,c). In the crosslinked blends (i.e., LDPE IR DCP and LDPE IR DCP Lig 5), some trenches were observed, indicating that high amounts of energy are needed to break the samples. During high shear melt-compounding and compression molding at elevated temperature (>150 °C), DCP molecules are cleaved and form free radicals, which abstract predominantly allylic hydrogen of polyisoprene and hydroxyl hydrogen of lignin, resulting in the crosslinked phase morphology in the LDPE/IR/Lignin blends. Such covalent crosslinking improved blend compatibility and thus increased melt-viscosity and other associated key mechanical properties of the blends.

**Figure 8:**
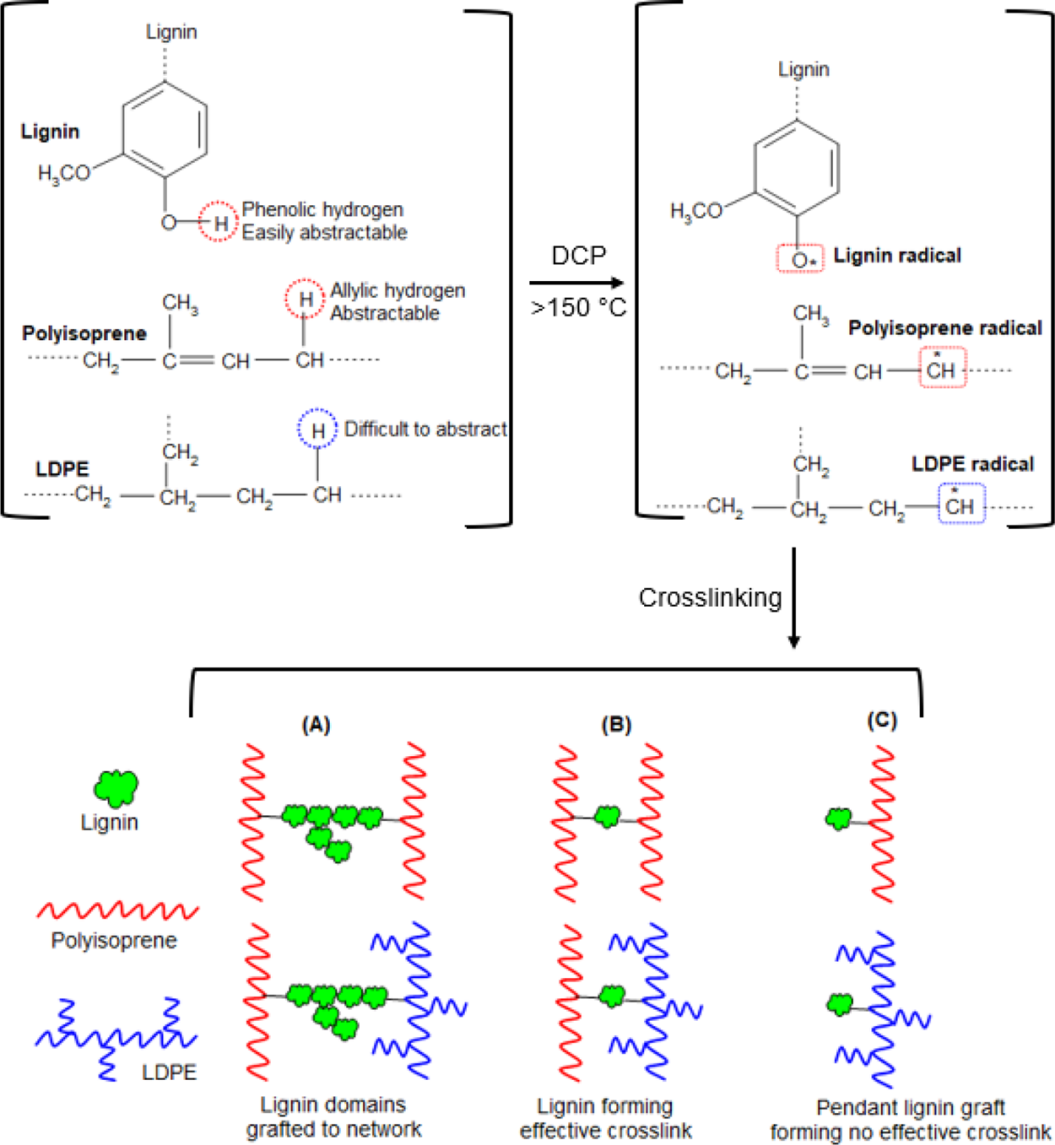
Possible peroxide crosslinking mechanism of the LDPE/IR blend involving lignin as a coagent.

Solubility study revealed that both neat and DCP crosslinked blends of LDPE/IR were disintegrated while immersed in the 1,2,4-trichlorobenzene solvent at 160 °C for 24 h, indicating that the blends were not strongly crosslinked like traditional thermoset materials. In contrast, the blends consisting of lignin (5 wt. % and more) showed the average insoluble mass residues (i.e., lignin + LDPE + IR) in the range of 28.8 to 36.4%, corresponding to the crosslinked LDPE/IR phases in the range of 22.2 to 25.1%, considering lignin is insoluble in the solvent. Notably, the blend with 5 wt. % lignin contained a more crosslinked LDPE/IR phase (25.1 wt. %) compared to the blends with 10 and 20 wt. % lignin, indicating excess lignin present in the blends interfere in the polymer crosslinking behavior (Table 1). However, it revealed that lignin acted as a cocuring agent and enhanced the crosslinking of lignin with LDPE/IR phases. It is well reported that the unsaturated polyisoprene phase is crosslinked preferentially instead of saturated polymers such as polyethylene when a peroxide curing system is used. Generally, organic peroxide results in slow curing rates and low crosslink densities and also deleterious side reactions such as polymer chain degradation. The addition of cocuring agents (coagents) based on multifunctional reactive organic molecules overcomes most of the deficiencies associated with peroxide cure systems.^41,60,61^ It is well documented that because of polyphenolic structures with hydrogen transfer or donating ability, lignin compounds are used as radical scavenging agents in the medical field.^43,44,62^ Therefore, it is anticipated that in the present study, lignin compounds with its hydroxyl groups can react with DCP radicals, forming lignin macroradicals. These lignin radicals react with LDPE/IR phases during high shear melt mixing at elevated temperatures. Therefore, lignin has the potential to tune the crosslinking mechanism in the LDPE/IR blends. At high temperatures, the homolytic cleavage of organic peroxide produces odd electron radical fragments, which can preferably abstract allylic hydrogen from unsaturated polyisoprene and hydroxyl hydrogen from lignin or undergo an addition reaction with the unsaturated double bonds of polyisoprene, resulting in the formation of highly reactive macro-free radicals. The polymer macroradicals produced by either abstraction or addition further undergo coupling reactions and form the C–C crosslink between the polymer chains. The possible crosslinking mechanism of LDPE/IR blends with DCP and lignin is presented in Figure 8. In such reactions, polyisoprene and lignin phases are crosslinked preferentially instead of polyethylene.

## 3 Experimental Section

### 3.1 Materials and the Blending Method

The LDPE resin with a melt flow index of 25 g/10 min (190 °C/2.16 kg) was obtained from Sigma Aldrich, Saint Louis, MO. This LDPE had a weight average molecular weight (*M*_w_) in the range of 135–163 kDa, as determined using a gel permeation chromatography system equipped with a light scattering detector. Dicumyl peroxide with a purity of 98% was obtained from Sigma Aldrich, Saint Louis, MO. NIPOL IR2200-grade polyisoprene rubber with a high cis-1,4 isoprene unit and a Mooney viscosity of 75–90 at 100 °C was obtained from Zeon Chemicals L.P., Louisville, KY. NIPOL IR2200 is identical to natural rubber while considering molecular structure and properties. Organosolv lignin isolated from hybrid poplar biomass was supplied by the American Science and Technology Inc., Wausau, WI. It had a purity of 93.4 wt. % with an average molecular mass of 2262 Da, and aliphatic OH groups of 4.4 mmol/g, phenolic OH groups of 3.1 mmol/g, and syringyl OH groups of 2.1 mmol/g.^28^

The blended materials were prepared according to the compositions shown in Table 1 using the IntelliTorque Plasticorder (Brabender CWB, South Hackensack, NJ) at the initial set temperature of 140 °C, a rotor speed of 90 rpm, and a mixing time of 30 min. The lignin content in the blends was varied from 0 to 20 wt. %. The mixing torque and stock temperature for each blend were recorded and discussed in this report. The blended samples were compression-molded at 170 °C and 5 MPa pressure for 10 min using a Carver press into films with the dimensions of 100 mm × 100 mm × 0.50 mm.

### 3.2 Materials Characterization

The tensile properties of the blends were measured using a universal testing machine (Instron 5943) according to the ASTM D-638 standard. A load cell of 10 kN, a gauge length of 15 mm, and an extension speed of 10 mm/min were used. Each test specimen had an average width of 4.00 mm and a thickness of 0.50 mm. An average of five repeat measurements per sample was obtained, and the data were analyzed using Microsoft Excel. The average values of tensile properties with standard deviations were reported. The DMA of the samples was carried out using DMA-850 (TA instruments) at the temperature ramp from −140 to 100 °C at a heating rate of 3 °C/min, an oscillation frequency of 0.1 Hz, and a strain of 0.1% in tensile mode. The melt-rheological characteristics to assess the thermal recyclability and the melt-flow behavior of the blends and individual polymers were investigated using a discovery hybrid rheometer (DHR-3, TA Instruments, New Castle, DE). The rheological measurements were performed under a nitrogen atmosphere using 25 mm diameter parallel plates with a sample gap of 1000 μm. Frequency sweeps were performed from 100 to 0.1 rad/s at 170 °C under 1% strain. The rheological analysis was performed using Trios software provided by TA Instruments.

The interfacial adhesion and the morphology of the blends were evaluated using the SEM technique. The cryogenically fractured cross-sectional surfaces of the materials were analyzed with a Quanta 650 FEG SEM, using a 5 kV beam voltage. Before analyzing, the samples were coated for 30 s in 50 mTorr argon with Au/Pd (ca. 6 nm coating) at 20 mA using a Denton Desk V sputter coater. The solubility and the extent of crosslinking of the materials were tested using 1,2,4-trichlorobenzene (Fisher Chemical) solvent. Three specimens of each sample with initial weights of approximately 100 to 150 mg were immersed in the solvent and conditioned at 160 °C for 24 h. As appropriate, the swelled materials were dried in a vacuum oven at 60 °C for 48 h to evaporate any residual solvent present in the samples. The average percentages of insoluble masses and crosslinked LDPE/IR phases were determined and reported in this study.

The differential scanning calorimetric studies of the samples (ca. 5 mg each sample) were carried out using a DSC 250 (TA Instruments, New Castle, DE) instrument under a nitrogen gas flow rate of 50 mL/min. The second heating and cooling cycles (temperature range of 30 to 250 °C) were recorded at a 10 °C/min rate and described in the report. Each sample was isothermally heated for 10 min at 250 °C before cooling. The DSC data were analyzed using Trios software provided by TA Instruments. The percentage of crystallinity values of the LDPE-based samples were calculated using the following equation:

percent (%) crystallinity 

Δ*H*_m_ is the experimentally obtained melting enthalpy value of the sample, (J g^–1^), (1 – α) is the weight percent of LDPE in the sample, and Δ*H*_m_^o^is the enthalpy value of melting of a 100% crystalline form of LDPE (293 J g^–1^).^63^

## 4 Conclusions

This study revealed a new direction of research on modulating the peroxide crosslinking behavior of plastic/rubber blends using the organosolv lignin as a cocuring additive to create TPEs. It is well documented in the rubber industries that organic peroxide can produce polymer chain crosslinking along with several competitive undesirable or destructive side reactions involving polymer chain scission or other degradation products, if there are fewer number of reactive sites on the polymer matrix. During thermomechanical processing, the polyphenolic lignin with its reactive OH groups can act as a hydrogen atom donor and produce lignin macroradicals. This lignin radical can behave as a coagent in improving effective crosslinking of the blends via establishing a higher concentration of reactive sites and reducing the chances of deleterious radical side reactions. In this study, the organosolv lignin was applied as a coagent additive to the blend of LDPE/polyisoprene crosslinked with DCP. The addition of lignin up to 5 wt. % to the LDPE/IR blends of 70/30 mass ratio, followed by vulcanization with 2 pph DCP, improved various properties of the blends including tensile strength and elongation. For example, the average values of the tensile strength, modulus, and ultimate elongation of neat LDPE resin were 7.8 MPa, 177 MPa, and 62%, respectively, and of LDPE/IR/lignin/DCP 65/30/05/2 were 8.1 MPa, 95 MPa, and 238%, respectively. It indicated that the application of lignin/DCP has a profound effect on improving ductility and elastomeric characteristics of the materials, and this material can have the potential to replace traditional thermoset rubbers. In addition, the lignin can contribute to reducing the melt-viscosity of crosslinked LDPE/IR blends because of the plasticization effect of the non-crosslinked lignin, which, in turn, facilitates the thermal reprocessing of the materials. It also appeared that phase morphologies of the modified blends, as seen in the electron microscopic imaging, are in harmony with the characteristics such as tensile, melt viscosity, and elasticity of the blends. In brief, organosolv lignin has good melt-flow properties and can disperse well within the thermoplastic matrix during melt-compounding, acting as an efficient cocuring agent during the vulcanization of LDPE/IR blends with organic peroxide.
